# An integrative approach for pediatric auditory neuropathy spectrum disorders: revisiting etiologies and exploring the prognostic utility of auditory steady-state response

**DOI:** 10.1038/s41598-020-66877-y

**Published:** 2020-06-17

**Authors:** Pei-Hsuan Lin, Chuan-Jen Hsu, Yin-Hung Lin, Yi-Hsin Lin, Shu-Yu Yang, Ting-Hua Yang, Pei-Lung Chen, Chen-Chi Wu, Tien-Chen Liu

**Affiliations:** 1 Graduate Institute of Clinical Medicine, National Taiwan University College of Medicine, Taipei, Taiwan; 2 Department of Otolaryngology, National Taiwan University Hospital Yunlin Branch, Yunlin, Taiwan; 3 Department of Otolaryngology, National Taiwan University Hospital, Taipei, Taiwan; 4 Department of Otolaryngology, Taichung Tzu-Chi Hospital, Taichung, Taiwan; 5 Graduate Institute of Medical Genomics and Proteomics, National Taiwan University College of Medicine, Taipei, Taiwan; 6 Department of Medical Genetics, National Taiwan University Hospital, Taipei, Taiwan

**Keywords:** Diseases, Medical research

## Abstract

Auditory neuropathy is an important entity in childhood sensorineural hearing loss. Due to diverse etiologies and clinical features, the management is often challenging. This study used an integrative patient-history, audiologic, genetic, and imaging-based approach to investigate the etiologies and audiologic features of 101 children with auditory neuropathy. Etiologically, 48 (47.5%), 16 (15.8%), 11 (10.9%), and 26 (25.7%) children were categorized as having acquired, genetic, cochlear nerve deficiency-related, and indefinite auditory neuropathy, respectively. The most common causes of acquired and genetic auditory neuropathy were prematurity and *OTOF* mutations, respectively. Patients with acquired auditory neuropathy presented hearing loss earlier (odds ratio, 10.2; 95% confidence interval, 2.2–47.4), whereas patients with genetic auditory neuropathy had higher presence rate of distortion product otoacoustic emissions (odds ratio, 10.7; 95% confidence interval, 1.3–85.4). In patients with different etiologies or pathological sites, moderate to strong correlations (Pearson’s *r* = 0.51–0.83) were observed between behavioral thresholds and auditory steady-state response thresholds. In conclusion, comprehensive assessments can provide etiological clues in ~75% of the children with auditory neuropathy. Different etiologies are associated with different audiologic features, and auditory steady-state responses might serve as an objective measure for estimating behavioral thresholds.

## Introduction

Auditory neuropathy is a challenging clinical disorder accounting for ~10% of cases of sensorineural hearing loss (SNHL) in children^1–3^. The pathogenesis of auditory neuropathy encompasses a wide range of disease mechanisms, with pathologies localized to multiple sites along the auditory pathway, including inner hair cells^4^, synapses^5^, spiral ganglion neurons^6^, auditory nerve^7^, or brainstem auditory nuclei^8^. Many acquired and genetic factors can contribute to auditory neuropathy^1,2,9,10^. Acquired auditory neuropathy include infection during pregnancy, prematurity, kernicterus, and perinatal hypoxia^1,9^. Auditory neuropathy may also be associated with certain types of syndromic or non-syndromic hereditary hearing impairment^9^.

Genetic factors may contribute to approximately 40% of auditory neuropathy patients in whom clinical manifestations might present as components of specific syndromes or as isolated non-syndromic disorders^9^. A number of hereditary neurodegenerative syndromes have been associated with auditory neuropathy, including Charcot-Marie-Tooth disease, Friedreich’s ataxia, Leber’s hereditary optic neuropathy, autosomal dominant optic atrophy, autosomal recessive optic atrophy, Mohr-Tranebjaerg syndrome, Refsum disease, Wolfram syndrome, Pelizaeus-Merzbacher disease, and CAPOS syndrome^9,11–14^. Genetic causes of non-syndromic auditory neuropathy include autosomal dominant mutations in *DIAPH3* and *PCDH9*; autosomal recessive mutations in *OTOF, PJVK*, and *GJB2*; and mitochondrial mutations in *MT-RNR1*^9,11^.

Audiologically, auditory neuropathy is characterized by mild to profound SNHL in the presence of otoacoustic emissions (OAEs) and/or cochlear microphonics (CMs), but absent or abnormal auditory brainstem responses (ABRs) and absent acoustic reflexes^2,9,10^. Given the heterogeneous etiologies and diverse audiologic features, benefits derived from hearing aids or cochlear implants (CIs) vary significantly among patients^2,10,15,16^. This poses difficulties in the management of auditory neuropathy. Recent advances in molecular genetics and imaging technologies have revolutionized diagnosis, counseling for, and treatment of childhood SNHL by precisely defining the etiology and pathology^17,18^. This is especially relevant for pediatric auditory neuropathy, as treatment outcomes correlate closely with the underlying etiologies^19^. To explore practical factors important for the guidance of assessment and management, we revisited etiologies and hearing profiles in a large auditory neuropathy cohort using an integrative audiologic, genetic, and imaging approach.

## Results

### Determination of etiologies

One-hundred-and-one children with auditory neuropathy, including 57 (56.4%) boys and 44 (43.6%) girls, were enrolled in this study. Among them, forty-eight patients (47.5%) were considered to have acquired auditory neuropathy, including 20 with prematurity, 5 with kernicterus, 5 with perinatal hypoxia, and 18 with two or more pre-/perinatal events (Table 1). All patients underwent genetic testing, and 16 (15.8%) patients were considered to have genetic auditory neuropathy, including 13 non-syndromic patients (12 with bi-allelic *OTOF* mutations and 1 with a homoplasmic mitochondrial m.1555 A > G mutation), 1 with Wolfram syndrome (*WFS1* p.A684V mutation), 1 with autosomal dominant optic atrophy (*OPA1* p.C472R mutation), and 1 with Pelizaeus-Merzbacher disease (*PLP1* duplication). Among the 12 patients with bi-allelic *OTOF* mutations, 4 were homozygotes for *OTOF* p.E1700Q mutations and 8 were compound heterozygotes (2 had p.E841K/p.E1700Q mutations, 1 had p.D1235fs/p.E1700Q mutations, 1 had p.R1344X/p.E1700Q mutations, 2 had p.E1700Q/p.R1735W mutations, 1 had p.E1700Q/p.H1779Y mutations, and 1 had p.E1700Q/p.R1856W mutations). Eleven (10.9%) patients were found to have cochlear nerve deficiency (CND) based on the imaging studies. In 26 (25.7%) of the 101 patients, the etiology of auditory neuropathy was indefinite.

**Table 1:** Etiologies of auditory neuropathy of all patients.

	N = 101	(%)
**Acquired, N** = **48 (47.5%)**		
Prematurity	20	19.8
Kernicterus	5	5
Perinatal hypoxia	5	5
Prematurity + perinatal hypoxia	13	12.9
Prematurity + kernicterus	2	2
Kernicterus + perinatal hypoxia	2	2
Prematurity + kernicterus + perinatal hypoxia	1	1
**Genetic, N** = **16 (15.8%)**		
*OTOF* mutations	12	11.9
Mitochondrial 12S rRNA mutation	1	1
Autosomal dominant optic atrophy	1	1
Pelizaeus-Merzbacher disease	1	1
Wolfram syndrome	1	1
**Cochlear nerve deficiency, N** = **11 (10.9%)**	11	10.9
**Indefinite, N** = **26 (25.7%)**	26	25.7

Forty-six (95.8%) patients with acquired auditory neuropathy had hearing loss at birth, which was a higher percentage of patients than those in the other three groups (Table 2, *p* = 0.001; acquired vs. others: odds ratio, 10.2; 95% confidence interval, 2.2–47.4). Notably, 5 of the 26 patients in the indefinite group reported a family history of childhood-onset SNHL, indicating that auditory neuropathy in these patients might also be related to genetic factors.

**Table 2:** Basic characteristics of all patients.

	Total, N = 101(%)	Acquired, N = 48 (%)	Genetic, N = 16 (%)	CND, N = 11 (%)	Indefinite, N = 26 (%)	*P* value
**Age at diagnosis**, Mean±SD (years)	1.2 ± 2.5	0.5 ± 1.1	2.1 ± 3.7	1.8 ± 4.8	1.7 ± 2.1	0.053^a^
**Follow-up periods**, Mean±SD (years)	4.4 ± 3.3	4.5 ± 3.1	5.2 ± 3.4	3.6 ± 2.2	4.2 ± 4.0	0.668^a^
**Sex**						0.984^b^
Male	57 (56.4)	28 (58.3)	9 (56.3)	6 (54.5)	14 (53.8)	
Female	44 (43.6)	20 (41.7)	7 (43.8)	5 (45.5)	12 (46.2)	
**Onset of hearing loss**						0.001^c^
At birth	82 (81.2)	46 (95.8)	11 (68.8)	9 (81.8)	16 (61.5)	
<10 years	18 (17.8)	2 (4.2)	5 (31.2)	1 (9.1)	10 (38.5)	
≥ 10 years	1 (1)	0 (0)	0 (0)	1 (9.1)	0 (0)	
**Family History**						0.052^c^
SNHL in childhood	9 (8.9)	1 (2.1)	2 (12.5)	1 (9.1)	5 (19.2)	
**Pathological site**						<0.001^c^
Presynaptic	19 (18.8)	6 (12.5)	13 (81.3)	0 (0)	0 (0)	
Postsynaptic	39 (38.6)	26 (54.2)	2 (12.5)	11 (100)	0 (0)	
Presynaptic + postsynaptic	17 (16.8)	16 (33.3)	1 (6.3)	0 (0)	0 (0)	
N/A	26 (25.7)	0 (0)	0 (0)	0 (0)	26 (100)	

*Abbreviation*: CND, cochlear nerve deficiency; SNHL, sensorineural hearing loss; N/A, not available.

^a^ANOVA test.

^b^Pearson Chi-square test.

^c^Fisher’s exact test.

Auditory neuropathy can be classified as presynaptic (i.e., lesions at inner hair cells or ribbon synapses) or postsynaptic (i.e., lesions central to the ribbon synapse in the auditory pathway) based on the site of pathology^19^. Reported causes of presynaptic auditory neuropathy include perinatal hypoxia, *OTOF* mutations, and mitochondrial mutations, while reported causes of postsynaptic auditory neuropathy include prematurity, kernicterus, autosomal dominant optic atrophy, Pelizaeus-Merzbacher disease, and CND^14,19^. In 75 patients with identifiable etiologies and risk factors, 19 could be considered to have presynaptic auditory neuropathy, and 39 have postsynaptic auditory neuropathy. The sites of pathology differed significantly among the four groups (*p* < 0.001), with genetic auditory neuropathy most likely to be presynaptic (81.3%) and CND-related auditory neuropathy exclusively associated with postsynaptic pathologies (100%).

### Audiologic features

Distortion product otoacoustic emissions (DPOAEs) were present in 61 of the 101 patients (60.4%) at the time of diagnosis of auditory neuropathy (Table 3). These patients included 27 (56.3%), 14 (87.5%), 6 (54.5%), and 14 (53.8%) individuals with acquired, genetic, CND-related, and indefinite auditory neuropathy, respectively. The presence rate of DPOAEs was significantly higher in patients with genetic auditory neuropathy than in those in the other three groups (genetic vs. others: odds ratio, 10.7; 95% confidence interval, 1.3–85.4). The follow-up period of DPOAEs was 2.0 ± 2.0 years on average, and there was no significant difference among the four groups (*p* = 0.16). During the follow-up period, DPOAEs disappeared in 15 (55.6%), 5 (35.7%), 5 (83.3%) and 2 (14.3%) patients in the acquired, genetic, CND-related, and indefinite groups, respectively. On the other hand, CMs were identified in 95 of the 101 patients (94.1%) at the time of the first visit. These patients included 48 (100%), 13 (81.3%), 10 (90.9%), and 24 (92.3%) individuals with acquired, genetic, CND-related, and indefinite auditory neuropathy, respectively, showing no significant differences among the four groups (*p* = 0.059). In the 6 patients who did not have CMs (n = 4) or in whom they were unavailable (n = 2), the presence of DPOAEs enabled the diagnosis of auditory neuropathy.

**Table 3:** Audiologic features of all patients (%).

	Total, N = 101(%)	Acquired, N = 48 (%)	Genetic, N = 16 (%)	CND, N = 11 (%)	Indefinite, N = 26(%)	*P* value
**Threshold**^a^, Mean±SD (dB)	65.8 ± 23.1	62.4 ± 22.2	60.5 ± 16.5	73.9 ± 27.4	72.3 ± 25.4	0.163^b^
**Hearing loss pattern**						0.347^c^
Stable	34 (33.7)	14 (29.2)	8 (50)	4 (36.4)	8 (30.8)	
Fluctuating	22 (21.8)	14 (29.2)	1 (6.3)	1 (9.1)	6 (23.1)	
Progressive	23 (22.8)	13 (27.1)	5 (31.3)	2 (18.2)	3 (11.5)	
Improving	4 (4)	1 (2.1)	2 (12.5)	0 (0)	1 (3.8)	
Others	18 (17.8)	6 (12.5)	0 (0)	4 (36.4)	8 (30.8)	
**Configuration**						0.658^c^
Flat	49 (48.5)	23 (47.9)	9 (56.3)	5 (45.5)	12 (46.2)	
Sloping	31 (30.7)	17 (35.4)	3 (18.8)	5 (45.5)	6 (23.1)	
Others	21 (20.8)	8 (16.7)	4 (25)	1 (9.1)	8 (30.8)	
**DPOAEs**						0.059^d^
Absent at diagnosis	37 (36.6)	20 (41.7)	1 (6.3)	4 (36.4)	12 (46.2)	
Present at diagnosis	61 (60.4)	27 (56.3)	14 (87.5)	6 (54.5)	14 (53.8)	
*Follow-up periods of DPOAEs, Mean* ± *SD (years)*	2.0 ± 2.0	2.4 ± 1.7	2.1 ± 2.4	1.2 ± 1.2	1.5 ± 2.4	0.16^b^
*Disappeared during follow-up*	27 (44.3)	15 (55.6)	5 (35.7)	5 (83.3)	2 (14.3)	0.013^c^
N/A	3 (3)	1 (2.1)	1 (6.3)	1 (9.1)	0 (0)	
**Cochlear microphonics**						0.059^c^
Absent	4 (4)^e^	0 (0)	2 (12.5)^e^	0 (0)	2 (7.7)^e^	
Present	95 (94.1)	48 (100)	13 (81.3)	10 (90.9)	24 (92.3)	
N/A	2 (2)^e^	0 (0)	1 (6.3)^e^	1 (9.1)^e^	0 (0)	

*Abbreviation*: CND, cochlear nerve deficiency; DPOAEs, distortion product otoacoustic emissions; N/A, not available.

^a^Four-frequency-average of behavioral thresholds at the age of diagnosis.

^b^ANOVA test.

^c^Fisher’s exact test.

^d^Pearson Chi-square test.

^e^The diagnosis of auditory neuropathy in these patients was based on the presence of DPOAEs.

There were no significant differences in the hearing thresholds, hearing loss patterns, or audiogram configurations among the four groups. Nineteen patients underwent cochlear implantation, including 11 patients with genetic causes (9 patients with *OTOF* mutations, 1 with Wolfram syndrome, and 1 with autosomal dominant optic atrophy), 2 patients with CND, and 6 patients with indefinite auditory neuropathy. The post-operative auditory and speech performance in the two CND patients were suboptimal, with Categories of Auditory Performance (CAP) score of 5 and Speech Intelligibility Rating (SIR) score of 1 in both patients, despite more than 3 years of rehabilitation. In contrast, all the other 17 patients without CND showed favorable outcomes with CI, with CAP scores of up to 6–7 and SIR scores of up to 3–5 within 3 years post-operatively.

### Imaging findings

**Figure 1:**
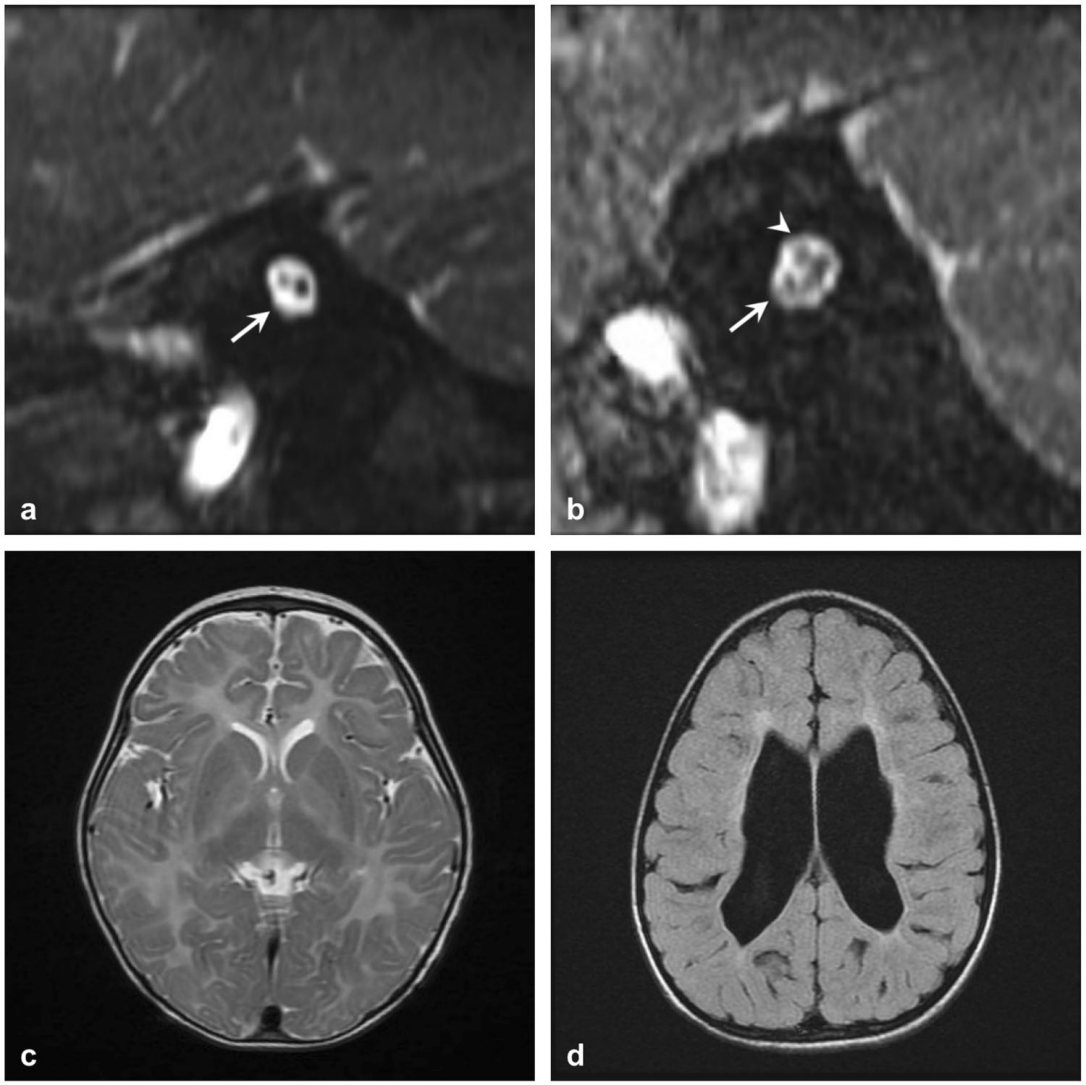
Representative imaging findings on posterior fossa magnetic resonance imaging T2-weighted images. (**a**) Sagittal view showing cochlear nerve aplasia. The arrow indicates the absence of the cochlear nerve. (**b**) Sagittal view showing cochlear nerve hypoplasia. The cochlear nerve (arrow) is thinner than the facial nerve (arrow head). (**c**) Axial view showing hypomyelination of the frontoparietal central parenchyma. (**d**) Axial view showing diffuse parenchymal loss.

The imaging findings are summarized in Table 4. CND was identified in 11 patients, including 8 with cochlear nerve aplasia (Fig. 1a) and 3 with cochlear nerve hypoplasia (Fig. 1b). Inner ear anomaly was identified in 1 patient with acquired auditory neuropathy (prematurity-related), who had dysplastic changes in the vestibule and semicircular canals. Abnormalities of the central nervous system were identified in 10 patients with acquired neuropathy, including 1 with cerebral hypomyelination (Fig. 1c), 1 with diffuse cerebral parenchymal loss (Fig. 1d), 5 with thin corpus callosum, and 3 with both cerebral hypomyelination and thin corpus callosum. Moreover, abnormalities of the central nervous system were identified in 1 patient with genetic auditory neuropathy (Pelizaeus-Merzbacher disease, cerebral hypomyelination), in 1 patient with CND (cerebral hypomyelination and thin corpus callosum), and in 1 patient with indefinite auditory neuropathy (cerebral hypomyelination).

**Table 4:** Imaging findings of all patients.

	Total, N = 83(%)	Acquired, N = 36(%)	Genetic, N = 14(%)	CND, N = 11(%)	Indefinite, N = 22(%)
Cochlear nerve deficiency	11 (13.3)	0 (0)	0 (0)	11 (100)	0 (0)
Inner ear malformations	1 (1.2)	1 (2.8)	0 (0)	0 (0)	0 (0)
Central nervous system abnormalities					
Cerebral hypomyelination	7 (8.4)	4 (11.1)	1 (7.1)	1 (9.1)	1 (4.5)
Diffuse parenchymal loss	1 (1.2)	1 (2.8)	0 (0)	0 (0)	0 (0)
Thin corpus callosum	9 (10.8)	8 (22.2)	0 (0)	1 (9.1)	0 (0)
**Total abnormalities**	**24 (28.9)**	**11 (30.6)**^**a**^	**1 (7.1)**	**11 (100)**^**b**^	**1 (4.5)**

*Abbreviation*: CND, cochlear nerve deficiency.

^a^Three patients with acquired auditory neuropathy presented both cerebral hypomyelination and thin corpus callosum.

^b^One patient with CND also had cerebral hypomyelination and thin corpus callosum.

### Correlations of Behavioral Thresholds and Auditory Steady-State Response (ASSR) Thresholds

**Figure 2:**
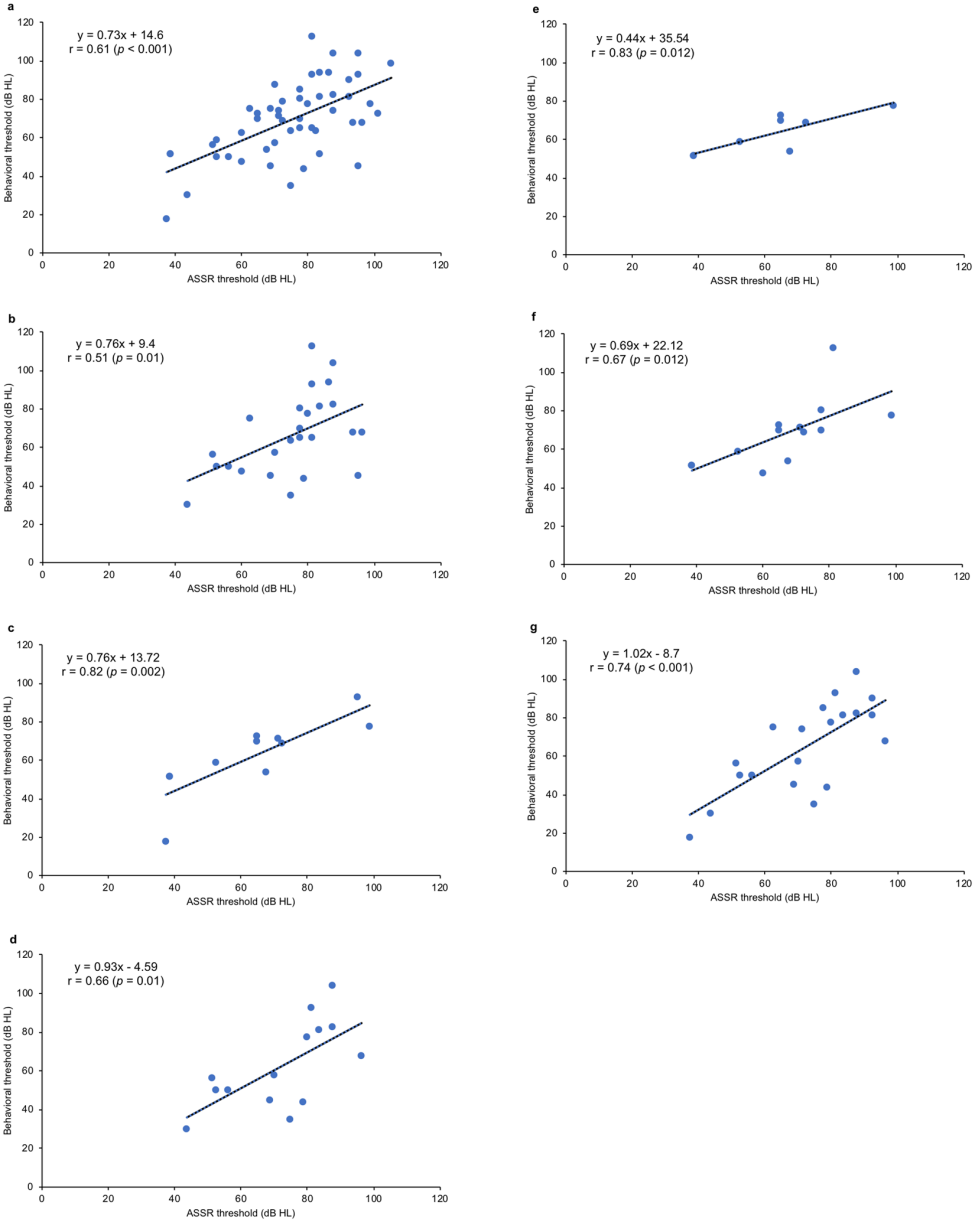
Correlations between behavioral thresholds and auditory steady-state response (ASSR) thresholds. (**a**) The correlation between behavioral and ASSR thresholds in all patients. (**b**,**c**) The correlations between behavioral and ASSR thresholds in patients with acquired and genetic auditory neuropathy, respectively, and representative behavioral and ASSR audiograms for the same patients from each group. (**d**,**e**) The correlations between behavioral and ASSR thresholds among prematurity-related auditory neuropathy patients and *OTOF*-related auditory neuropathy patients, respectively. (**f,g**) The correlations between behavioral and ASSR thresholds in patients with presynaptic and postsynaptic auditory neuropathy, respectively.

To objectively estimate hearing levels in the patients with auditory neuropathy, we compared the data obtained from different audiologic tests. We calculated the correlations between the thresholds of behavioral audiograms (i.e. visual reinforcement audiometry, conditioned play audiometry, and pure tone audiometry) and ASSR measured at the same testing time. The data were available to be analyzed in a total of 51 patients. These patients were tested at the age of 6 months to 7 years old, with 48 of them (94%) tested at younger than 3 years of age. Pearson correlation analyses revealed a moderate positive correlation between the behavioral and ASSR thresholds (*r* = 0.61, *p* < 0.001; Fig. 2a). The slope of the regression line between the behavioral and ASSR thresholds (0.73), however, did not approach 1, indicating that correction is required to estimate behavioral thresholds based on ASSR thresholds.

When classified according to the etiology, there was a moderate correlation between the behavioral and ASSR thresholds (*r* = 0.51, *p* = 0.01, slope = 0.76; Fig. 2b) in patients with acquired auditory neuropathy, whereas there was a strong correlation between the behavioral and ASSR thresholds (*r* = 0.82, *p* = 0.002, slope = 0.76; Fig. 2c) in patients with genetic auditory neuropathy. Specifically, there was a moderate correlation between the behavioral and ASSR thresholds (*r* = 0.66, *p* = 0.01, slope = 0.93; Fig. 2d) in patients with prematurity-related auditory neuropathy, and strong correlation between the behavioral and ASSR thresholds (*r* = 0.83, *p* = 0.012, slope = 0.44; Fig. 2e) in patients with *OTOF* mutations.

Similarly, when classified according to the site of pathology, we also observed moderate correlations between behavioral and ASSR thresholds both in patients with presynaptic auditory neuropathy (*r* = 0.67, *p* = 0.012, slope = 0.69; Fig. 2f) and those with postsynaptic auditory neuropathy (*r* = 0.74, *p* < 0.001, slope = 1.02; Fig. 2g).

## Discussion

Despite the etiological heterogeneity, we were able to obtain information regarding the cause of pediatric auditory neuropathy in ~75% (75/101) of the patients using comprehensive history-taking, genetic examinations, and imaging studies. The major factors related to auditory neuropathy were acquired factors (i.e., pre-/perinatal events), followed by genetic and developmental (e.g., CND) factors. Prematurity, either isolated or in combination with other risk factors, was the most commonly identified pre-/perinatal event contributing to acquired auditory neuropathy in this study. Selective loss of inner hair cells and relative preservation of outer hair cells have been reported as causes of auditory neuropathy in premature infants^4,20^. In addition, prematurity is associated with several comorbid factors including hyperbilirubinemia and hypoxia, which might also contribute to auditory neuropathy^2,21^.

Secondary to the acquired factors, genetic etiologies constituted another important common cause of auditory neuropathy in our cohort. Recessive *OTOF* mutations were the predominant genetic cause among patients with non-syndromic auditory neuropathy in this study. Excluding the 48, 3 and 11 patients who were confirmed to have acquired, syndromic auditory neuropathy, and CND-related auditory neuropathy, respectively, recessive *OTOF* mutations accounted for 12 (30.8%) of the remaining 39 patients with non-syndromic non-acquired auditory neuropathy. This prevalence is similar to those reported in previous studies in other populations, wherein *OTOF* mutations were detected in approximately 30–50% of patients with non-syndromic auditory neuropathy^22–28^.

CND was detected using imaging studies in 11 of the patients without identified acquired or genetic factors. CND may be unilateral or bilateral and may occur in children with physiologically normal cochleae^29,30^. As a well-documented imaging abnormality associated with auditory neuropathy and poor CI outcomes, the pathogenesis of CND in humans remains largely unclear. CND may result from the nerve either partially (hypoplasia) or completely (aplasia or agenesis) development failure or as a consequence of post-developmental degeneration^29^. Animal studies indicate that the expression of certain neurotrophic factors such as brain-derived neurotrophic factor and neurotrophin-3 by the otocyst is required for early cell migration, neurite outgrowth, and survival of vestibular and cochlear ganglion cells^31,32^. Absence of these factors might lead to the loss of ganglion cells and agenesis of the cochlear nerve^31,32^.

While clarifying the pathology of the disease is crucial for designing the treatment plan, some patients may have more than one pathological site. For instance, certain disease entities, such as Wolfram syndrome, could involve both presynaptic and postsynaptic pathologies^33^. In addition, some patients could have more than one risk factors for auditory neuropathy at birth, such as having both kernicterus and perinatal hypoxia, which might also result in presynaptic and postsynaptic lesions simultaneously. Accordingly, among the 75 patients with identifiable etiologies and/or risk factors, the pathological sites could not be ascertained in 17 patients, as presynaptic and postsynaptic lesions might coexist. More sophisticated investigations, such as electrocochleography, are needed to delineate the actual pathological sites of auditory neuropathy in these patients.

Our results indicate that patients with auditory neuropathy with distinct etiologies exhibited different hearing profiles. For instance, more than 95% of patients with acquired auditory neuropathy presented with symptoms at birth immediately after pre-/perinatal insults, whereas about 30–40% of patients with genetic and indefinite causes developed auditory neuropathy later in life. Late-onset progressive auditory neuropathy has been reported in patients with *OTOF* or *OPA1* mutations^34,35^, this is consistent with our observation. In addition, the presence rate of DPOAEs was higher in patients with genetic auditory neuropathy than in those in the other 3 groups. This might be attributed to the high percentage of patients with *OTOF* mutations in the genetic group. As the pathology of *OTOF* mutations is confined to the ribbon synapses of inner hair cells, the high presence rate of DPOAEs might reflect the relatively intact function of outer hair cells.

On the other hand, most of the patients presented with CMs, which was consistent among the four groups. In contrast to OAEs, which might disappear with time in patients with auditory neuropathy^36,37^, CMs usually remain stable with time^38^. Absent OAEs have been reported in some patients with auditory neuropathy despite the presence of CMs^1,39^. It has been postulated that in such patients, CMs are generated in both outer and inner hair cells, and that the outer hair cells are able to produce CMs, but are sufficiently impaired so as to fail in the generation of OAEs^1^.

Patients with auditory neuropathy may exhibit a range of hearing thresholds on subjective audiologic tests, ranging from normal to profound hearing loss, and hearing levels may change at different measurements^1,2,19^. Moreover, objective audiologic tests commonly used to estimate behavioral thresholds, such as the ABR and acoustic reflex, are of limited utility in children with auditory neuropathy due to the absence of responses in such patients. It is thus difficult to determine the appropriate amplification level when using hearing aids. In this study, we investigated the usefulness of ASSR to estimate the hearing levels in auditory neuropathy patients. Although ASSR has been confirmed as a reliable tool for the estimation of behavioral thresholds in cochlear SNHL^40,41^, previous studies on its utility in patients with auditory neuropathy have had inconsistent results. Some studies failed to find significant correlations between behavioral and ASSR thresholds^41,42^, whereas others have suggested that ASSR might serve as a valuable objective measure of behavioral thresholds in children with auditory neuropathy (e.g., Attias, *et al*.^43^). Our correlation analyses in a relatively large cohort of patients with pediatric auditory neuropathy revealed moderate to strong correlations (*r* = 0.51–0.83) between behavioral and ASSR thresholds in patients with different etiologies or sites of pathology. As such, in the absence of other reliable objective audiologic tests, the results of ASSR might offer some insights into hearing acuity in children with auditory neuropathy.

In addition to achieving a high diagnostic yield rate in the etiologic work-up, the integration of genetic and imaging studies into clinical assessments also had advantages for decision-making in the management of auditory neuropathy. The anatomic location of the pathology has been identified as a variable predictive of CI performance^44,45^, and both genetic and imaging studies are useful in pinpointing the site of pathology in auditory neuropathy. For instance, previous studies reported that patients with auditory neuropathy with *OTOF* mutations usually had excellent CI outcomes, likely because the pathology is confined to the synapse, and postsynaptic neurons and nerve fibers are intact for electrical stimulation of CI^46–48^. Cochlear implantation should thus be performed in patients with auditory neuropathy with *OTOF* mutations whenever indicated without unnecessary delay to achieve better CI outcome^49^. On the other hand, the presence of CND on imaging studies is indicative of unfavorable CI performance^50^. Although children with CND who have small nerves may benefit from cochlear implantation or amplification, such interventions are contraindicated in children with completely absent cochlear nerves^51^.

The major strength of this study lies in the comprehensive etiologic and audiologic analyses carried out in a large cohort of patients with pediatric auditory neuropathy. However, some limitations merit discussion. First, biases inherent in the retrospective nature of the study could have influenced our results. For instance, we only have limited data in our patients regarding the newborn hearing screening (NHS) results, speech perception, and language development. Many of our patients were diagnosed with auditory neuropathy before the implementation of the universal newborn hearing screening program by the Taiwan government in 2012. Patients who did not receive NHS and those who received NHS using OAEs might have delayed diagnosis of auditory neuropathy. Similarly, given the relatively young age of our patients and the compromised speech perception associated with auditory neuropathy, we were unable to obtain sufficient data on speech perception and language development for further analyses. Second, because our analyses focused only on children with auditory neuropathy, our findings cannot be generalized to adult patients with auditory neuropathy. Finally, although our results demonstrated some utility of ASSR in estimating the hearing level in pediatric patients with auditory neuropathy, the estimation was not optimal, as only moderate to strong correlations were present between behavioral and ASSR thresholds. Exploration and validation of other objective audiologic tests are thus warranted. Cortical auditory evoked potentials may be a potential candidate, as young patients with auditory neuropathy generally have recordable cortical potentials despite absent or abnormal brainstem responses^52,53^.

In conclusion, comprehensive history-taking, genetic examinations, and imaging studies can provide etiological clues in approximately 75% of children with auditory neuropathy. Different etiologies are associated with different audiologic features, likely due to different anatomic sites of the pathologies involved. In the absence of other reliable objective audiologic tests, ASSR might serve as an objective measure for the estimation of behavioral thresholds.

## Materials and Methods

### Patient recruitment

From 1997–2018, children diagnosed with bilateral auditory neuropathy at a tertiary university-affiliated medical center were included for the study. Diagnosis of auditory neuropathy was based on repeated findings of absent or abnormal ABR waveforms, and the presence of OAEs or CMs. Basic demographic data, family history, maternal history, birth history, and past medical history were ascertained for each patient. Patients older than 18 years, those with conductive or mixed-type hearing impairment, those with unilateral hearing impairment, and those without complete history records were excluded. This study was approved by the Research Ethics Committee of the National Taiwan University Hospital. Written informed consent was obtained from all patients and/or their parents. All methods were performed in accordance with the relevant guidelines and regulations.

### Audiologic tests

Each patient underwent a battery of audiologic assessments appropriate for his/her age and neurological status, including DPOAEs, ABR, ASSR, and behavioral audiometries. The assessments were performed at least once every 6 months during the follow-up periods. DPOAEs were analyzed by using Titan – DPOAE440 (Interacoustics, Middelfart, Denmark). The f2/f1 ratio was 1.22, and the intensity ratio was set at 65/55 dBSPL. The frequency settings were 1, 1.5, 2, 3, 4, and 6 kHz. For each frequency, a signal-to-noise ratio of> 6 dB was determined as a positive DPOAEs response, and at least five frequencies with a positive DPOAEs response were considered to have presence of OAEs. The CMs were identified using ABR clicks at 95 dBnHL by comparing different types of stimulus polarities (rarefaction, condensation, and alternating). Additional control tests with the tubing clamped under same stimulus were applied to confirm the presence of artifacts. ASSR was performed with Bio-logic Multiple Auditory Steady-state Response (MASTER) II software system (Natus Medical Inc., IL, USA) running on a Navigator PRO hardware platform (Natus Medical Inc., IL, USA). The patients were either naturally asleep or sedated with chloral hydrate. Each ear was tested for four frequencies, including 0.5, 1, 2, and 4 kHz, simultaneously, and the modulation frequencies were set between 82–99 Hz with different carrier frequencies at both sides. Behavioral audiometries were performed using visual reinforcement audiometry, conditioned play audiometry, or pure tone audiometry, depending on the developmental level of the patient.

The audiologic data included hearing levels, onset of hearing loss, hearing loss patterns, and configurations of audiograms^54,55^. Hearing levels were determined by the four-frequency average thresholds (0.5, 1, 2, and 4 kHz). Hearing loss onset was categorized as at birth (i.e., those who failed newborn hearing screening), younger than 10 years, and 10 years or older. At least three consecutive audiologic tests during at a follow-up period were ascertained to determine the hearing loss patterns, which were categorized as stable, fluctuating, progressive, improving, or other. The audiogram configurations were classified as flat, sloping, or other^54,55^. The CAP score and SIR scale were evaluated in patients who received cochlear implantation, before and after the operation, by a questionnaire provided to the parents during follow-up to evaluate the outcomes of hearing performance^46,56^.

### Genetic examinations

Genetic counseling was offered to all patients and/or their parents at the clinic. All subjects with informed consent for genetic testing had Sanger sequencing performed for four common deafness genes: *GJB2* (Gene ID: 2706), *SLC26A4* (Gene ID: 5172), *OTOF* (Gene ID: 9831), and the mitochondrial 12S rRNA gene (*MTRNR1*, Gene ID: 4549)^34,57^. Patients with bi-allelic *GJB2*, *SLC26A4* or *OTOF* mutations, and those with homoplasmic or heteroplasmic mitochondrial mutations were considered to have a definite genetic diagnosis. Patients in whom we were unable to identify common mutations underwent comprehensive genetic examinations using a next-generation sequencing panel targeting 159 known deafness genes^58–60^.

### Imaging studies

Non-contrast brain magnetic resonance imaging (MRI) with a resolution of 0.5-mm thickness was performed to investigate the central auditory pathway and cochlear nerve. Temporal bone high-resolution computed tomography (HRCT) with contiguous axial and coronal sections of 0.6-mm thickness was performed to investigate the structure of the inner ear. All images were reviewed according to previously published criteria^61–64^. CND was defined as a cochlear nerve diameter smaller than that of the facial nerve on the oblique sagittal MRI view of the internal auditory canal, or as a cochlear aperture smaller than 1.5 mm on an axial HRCT view^61,63,64^. Cerebral and brainstem abnormalities were determined with reference to the patient’s age^62^.

### Patient grouping

The patients were divided into the following four groups based on medical history, genetic testing results, and imaging findings: genetic, acquired, CND-related, and indefinite. Patients documented to have prenatal or perinatal events, including prematurity of less than 34 weeks of gestational age, history of kernicterus with plasmapheresis, and perinatal hypoxia were considered to have acquired auditory neuropathy^2,3,21^. Patients with genetic mutations and those with phenotypes related to specific hereditary syndromes were considered to have genetic auditory neuropathy. Patients without identified acquired or genetic factors who had CND based on imaging studies were considered to have CND-related auditory neuropathy. Other patients were considered to have auditory neuropathy of indefinite causes. The hearing profiles and imaging findings were then compared among the four groups.

### Statistical analysis

Statistical analyses were performed using SPSS for Windows version 22.0 (SPSS Inc., Chicago, IL, USA). Categorical data were analyzed using Pearson Chi-square test or Fisher’s exact test and summarized as number (%); continuous data were analyzed using ANOVA and summarized as mean ± standard deviation (SD). Pearson correlation analyses were performed to calculate correlations between hearing levels obtained using different audiologic tests. *P* values <0.05 (2-sided) were interpreted as statistically significant.

## Data Availability

The datasets generated during and/or analyzed during the current study are available from the corresponding authors on reasonable request.
